# Predictive Effects of Waist Circumference-Related Anthropometric Measures on Body Mass Index in South African University Students

**DOI:** 10.3390/ijerph23030385

**Published:** 2026-03-18

**Authors:** Howard Gomwe, Lesego Phiri, Chioneso Show Marange, Tandi Matsha, Mpho Kgoele

**Affiliations:** 1 Department of Physiotherapy, Faculty of Health Sciences, Sefako Makgatho Health Sciences University, Pretoria 0204, South Africa; 2 Skills Centre, Faculty of Health Sciences, Sefako Makgatho Health Sciences University, Pretoria 0204, South Africa; lesego.phiri@smu.ac.za; 3 Department of Computational Sciences, Faculty of Science and Agriculture, University of Fort Hare, East London 5201, South Africa; cmarange@ufh.ac.za; 4 Office of the Vice Chancellor, Sefako Makgatho Health Sciences University, Pretoria 0204, South Africa; tandi.matsha-erasmus@smu.ac.za; 5 Department of Nursing Science, Sefako Makgatho Health Sciences University, Pretoria 0204, South Africa; mpho.kgoele@smu.ac.za

**Keywords:** body mass index, waist circumference, waist-to-height ratio, waist-to-hip ratio

## Abstract

**Highlights:**

**Public health relevance—How does this work relate to a public health issue?**

- This study addresses the global and national public health issue of rising obesity, particularly among young adults, by highlighting the limitations of relying solely on Body Mass Index (BMI) for risk assessment.
- It focuses on a key demographic, that is, university students who are in a transitional life stage where early identification of unhealthy weight gain and central adiposity can inform timely public health interventions to prevent long-term cardiometabolic diseases.

**Public health significance—Why is this work of significance to public health?**

- The study provides evidence from an African context, where data is often lacking, supporting the use of simple, low-cost measures like Waist-to-Height Ratio (WHtR) and Waist Circumference (WC) as more robust indicators of health risk than BMI alone.
- It demonstrates that these central adiposity indicators are especially predictive at higher BMI levels, offering a more precise tool for identifying individuals within the student population who are at the greatest risk for future obesity-related diseases, enabling more targeted resource allocation.

**Public health implications—What are the key implications or messages for practitioners, policy makers and/or researchers in public health?**

- For practitioners: Routine health assessments for young adults should incorporate WHtR or WC alongside BMI to better identify individuals with adverse fat distribution and elevated cardiometabolic risk, leading to earlier lifestyle counseling and intervention.
- For policy makers & researchers: Public health screening guidelines and wellness programs in higher educational institutions should be updated to include measures of central adiposity. Researchers should prioritize longitudinal studies to confirm these indicators′ power in predicting actual disease outcomes in young African populations.

**Abstract:**

**Background:** Body mass index (BMI) is commonly used to assess obesity but does not differentiate between fat and lean mass, limiting its effectiveness to assess cardiometabolic risk. Measurements of central adiposity, such as waist circumference (WC), waist-hip ratio (WHR) and waist-to-height ratio (WHtR), are better predictors of metabolic dysfunction, especially with respect to visceral fat. **Aim:** To examine BMI trends and assess the association between BMI and waist-related anthropometric indicators WC, WHR, and WHtR, among university students in South Africa. **Methods:** A cross-sectional study involving 842 university students was conducted. Anthropometric measurements were taken according to ISAK standards. Quantile regression models supplemented by ROC curves were used to assess the predictive effects of WC, WHR, and WHtR on BMI across its distribution. **Results:** A total of 842 participants (63.8% female) were included, with a mean age of 21.8 ± 4.0 years. Significant differences were observed across BMI categories for all demographic and anthropometric characteristics (*p* < 0.001). Quantile regression analyses showed that WHtR and WC were strong and consistent predictors of BMI in all quantiles, with effect sizes increasing at higher levels of BMI. The WHR showed weaker associations overall, but these became significant in the upper BMI quantiles. Collectively, waist-related indicators, particularly WHtR and WC, exhibited robust predictive values for elevated BMI. **Conclusions:** Waist-related indicators, especially WHtR and WC are robust predictors of elevated BMI among university students, particularly in higher BMI ranges. These findings highlighted the value of incorporating central adiposity measures alongside BMI for more accurate health risk assessments in young adult populations.

## 1. Introduction

Assessing health risks using anthropometric measurements involves using simple physical measurements like height, weight, and body circumference to check a person’s body composition and possible health problems [1,2]. These measurements provide important insights into a person’s nutritional health status, fat distribution, such as subcutaneous and visceral fat, and overall health risk. To stay healthy, it is important to understand and regularly track these key body measurements [3,4]. According to the World Health Organization, obesity is usually measured using body mass index (BMI). BMI is widely used to assess the extent and severity of excess body fat in adolescents and adults by correlating weight with height. It is calculated by dividing a person’s weight in kilograms by the square of their height in meters. On the other hand, obesity is defined as an excessive accumulation of fat within the body’s adipose tissue. It is recognized as the most prevalent nutritional disorder among individuals in wealthy societies. Medically, obesity occurs when body fat increases to a level that may negatively affect overall health [5]. 

Physical inactivity, excessive calorie consumption, genetic predisposition, and endocrine abnormalities are among the common causes of obesity [6]. Obesity has now reached epidemic proportions worldwide, affecting not only developed countries but also developing ones. South Africa has the highest obesity rates on the African continent [7]. Like many developing countries, the nation faces a dual burden of malnutrition, where rising obesity levels coexist with ongoing undernutrition [8,9]. The increasing prevalence of lifestyle-related diseases further aggravates this already complex public health issue. For university students, the changes from adolescence to adulthood are often accompanied by substantial lifestyle adjustments [10,11]. Changes in diet, genetic predispositions, age-related changes, gender, and physical inactivity can influence overweight and obesity in adolescents [12,13,14,15].

Overweight is defined as a condition in which an individual’s body weight surpasses the recommended range for their age and height. Underweight, on the other hand, is defined as a lack of sufficient body weight; it is usually associated with deficient nutritional reserves in the body and a high risk of mortality in times of ill-health [16]. Based on BMI classification, a BMI below 18.5 kg/m^2^ is considered underweight, while a BMI between 18.5 and 24.9 indicates normal weight. A BMI from 25.0 to 29.9 is considered overweight, and a BMI of 30.0 or above is classified as obese [17,18,19].

Individuals classified as Class I obesity (BMI 30.0–34.9 kg/m^2^) are at an increased risk for developing cardiovascular disease, type 2 diabetes, obstructive sleep apnea, and several types of cancer [20]. Class II obesity (BMI 35.0–39.9 kg/m^2^) presents substantially higher risks of serious morbidity, including heart disease, stroke, and type 2 diabetes [21]. Lastly, Class III obesity (BMI ≥ 40.0 kg/m^2^) is linked to significantly elevated risks of severe health issues such as coronary heart disease, stroke, diabetes, and multiple obesity-related cancers [20,21]. Despite its common use in classifying obesity, BMI fails to distinguish between fat mass and lean body mass, which limits its accuracy in assessing cardiometabolic risk [22,23,24].

Research indicates that fat accumulation around the abdomen, referred to as central adiposity, presents a higher risk to health than general obesity measured by BMI [12]. Therefore, it is advisable to include waist circumference (WC), waist-to-hip ratio (WHR), and waist-to-height ratio (WHtR) as complementary indicators of central fat when assessing health risks, alongside BMI, to achieve a more accurate classification [25,26]. Waist circumference and WHR have commonly been employed as indicators of central obesity. More recently, WHtR has gained attention as a measure of central (visceral) adiposity and is increasingly recognized as an indicator of early health risk [27]. These indicators, WC, WHR, and WHtR, offer valuable insights into central obesity and body fat distribution. However, although numerous studies have examined the association between these body composition metrics and mortality, findings remain inconsistent and subject to further research [28].

These indicators provide additional insights into central or abdominal obesity, which has been strongly associated with cardiometabolic diseases such as type 2 diabetes and hypertension [29]. However, despite growing evidence of their potential value, the degree to which these measures can accurately predict, enhance, or complement BMI in evaluating overall health risk remains a topic of ongoing research. Therefore, exploring the association between BMI and more direct measures of overweight and obesity, such as WC, WHR, and WHtR, is essential for a more accurate and more comprehensive understanding of their health status [30,31,32]. There is a lack of information on the anthropometric data for predicting the health status of the population, especially from Africa [33]. This study examined the trend in BMI and the association between BMI and waist circumference-related measurements amongst the university students.

## 2. Methods

### 2.1. Design

A cross-sectional study was conducted to collect data on anthropometric measurements among university students in South Africa.

### 2.2. Sampling and Setting

The ethical clearance approval period by the ethics committee of the hosting university was from 1 December 2022 to 1 December 2023. The research study was conducted from 15 May to 30 October 2023. The study utilized voluntary response sampling combined with random selection to choose participants from a pool of university students who responded to an open call for participation. Invitations were sent through various channels, including university email, lecture announcements, and student organization platforms, to ensure a broad and diverse reach. Once expressions of interest were collected, all eligible respondents were assigned unique identification numbers. A simple computer-generated random sampling procedure was then used to select the final sample from this pool, ensuring that each respondent had the same chance of being included. This approach minimized selection bias and improved the representativeness of the sample. To facilitate easy access for students, the researchers set up gazebo tents around the university campus. Written informed consent to participate was obtained from all participants in the study.

### 2.3. Measures

The participants were asked to specify their age, gender and residential setting, indicating whether they lived on or off campus. The key anthropometric measurements required included height (cm), weight (cm), WC (cm), and hip circumference (cm). Measurements were obtained using methods established by the International Society for the Advancement of Kinanthropometry (ISAK). The measurements collected were then used to calculate the WHR and WHtR. The WHR was determined by dividing the WC by hip circumference, while the WHtR was obtained by dividing the WC by height. 

### 2.4. Statistical Analysis

All statistical analyses were performed using IBM SPSS Statistics Version 29 and RStudio version 2025.05.1+513. Descriptive statistics were computed to summarize the demographic and anthropometric characteristics of the study participants. Continuous variables were expressed as means and standard deviations (SD), while categorical variables were summarized using frequencies and percentages. Comparisons of demographic and anthropometric characteristics across BMI categories were conducted using one-way analysis of variance (ANOVA) for continuous variables and the Chi-square test for categorical variables. Significant ANOVA results were followed by Tukey’s HSD post hoc test. To examine associations between waist-related anthropometric indicators and BMI across the BMI distribution, quantile regression analyses were carried out at the 10th, 25th, 50th, 75th, and 90th percentiles. This approach allowed for the estimation of the differential effects of WC, WHR, and WHtR on BMI at various points in its conditional distribution. Unadjusted and adjusted models were estimated. The adjusted models included age and gender as covariates to account for their potential confounding effects. Regression coefficients with corresponding 95% confidence intervals (CIs) and *p*-values were reported. The quantile regression models were estimated under minimal distributional assumptions. Independence of observations and absence of multicollinearity were assessed, and model specification was evaluated through inspection of coefficient patterns across quantiles. To supplement the quantile regression findings, ROC curves were used to assess the discriminatory performance of waist circumference, waist-to-hip ratio, and waist-to-height ratio in relation to BMI. The area under the curve (AUC) with 95% confidence intervals was calculated for each measure, and statistical significance was evaluated relative to an AUC of 0.5. ROC analyses were conducted using a two-sided significance level of *p* < 0.05.

## 3. Results

Table 1 presents the demographic characteristics and waist-related anthropometric measurements of the study participants. A total of 842 individuals were included in the analysis, comprising 537 females (63.8%) and 305 males (36.2%). The mean age of the sample was 21.78 ± 4.04 years, indicating a predominantly young adult population. Participants had an average weight of 66.42 ± 14.94 kg and a mean height of 166.45 ± 9.19 cm. Regarding adiposity-related indicators, the mean BMI was 24.0 ± 5.3 kg/m^2^. The classification of the BMI categories showed that 8.3% of participants were underweight, 56.9% were of normal weight, 22.8% were overweight, and 12.0% were obese. The mean gluteal circumference was 102.37 ± 12.22 cm, while the average WC measured 79.85 ± 11.24 cm. Central adiposity indices showed a mean WHR of 0.78 ± 0.08 and a mean WHtR of 0.48 ± 0.07.

Table 2 summarizes the comparisons and associations between demographic characteristics, waist-related anthropometric measures, and BMI categories. Statistically significant differences were observed across all characteristics evaluated (*p* < 0.001 for most variables), indicating strong associations between BMI status and both demographic and central adiposity indicators.

The gender distribution differed significantly across BMI categories (*p* < 0.001). Among males, the majority were classified as normal weight (62.0%), while smaller proportions fell into the overweight (20.3%) and obese (6.2%) categories. In contrast, a larger proportion of females were classified as overweight (24.2%) or obese (15.3%), demonstrating a higher prevalence of elevated BMI among female participants.

Age increased progressively with BMI category, with mean ages ranging from 21.03 ± 3.63 years among underweight individuals to 23.28 ± 4.04 years among those classified as obese (*p* < 0.001). Statistically significant associations for weight and height were also observed. Weight increased with BMI, while height demonstrated an inverse pattern, with a lower average height recorded among overweight and obese participants (*p* < 0.001).

Central adiposity indicators showed strong positive associations with BMI. Gluteal circumference increased steadily across BMI groups, from 87.86 ± 5.25 cm in the underweight group to 121.67 ± 11.62 cm in the obese group (*p* < 0.001). Similar patterns were observed for WC, which increased from 70.30 ± 7.61 cm among underweight individuals to 97.26 ± 10.98 cm among those with obesity (*p* < 0.001). Waist-to-height ratio demonstrated the steepest gradient, ranging from 0.41 ± 0.04 in the underweight category to 0.60 ± 0.06 in the obese category, again with highly significant differences (*p* < 0.001).

Waist-to-hip ratio also differed significantly across BMI categories (*p* = 0.002), although the magnitude of differences was smaller compared to other adiposity indices. WHR values were similar in the normal weight and overweight groups (0.78 ± 0.07 and 0.78 ± 0.08, respectively) but higher among the underweight and obese groups (0.80 ± 0.09 and 0.80 ± 0.08, respectively).

Table 3 presents the results of unadjusted and adjusted quantile regression models assessing the predictive effect of WC, WHR, and WHtR on BMI across different quantiles (10th, 25th, 50th, 75th, and 90th percentiles). The adjusted model accounts for age and gender. Model fit and predictive performance were evaluated using pseudo-R^2^ and mean absolute error (MAE), respectively, for each estimated quantile.

In terms of model fit, WC and WHtR, pseudo-R^2^ values increased progressively across higher BMI quantiles, indicating greater explanatory power at the upper tail of the BMI distribution. This pattern was accompanied by generally lower MAE values at the median and upper quantiles, suggesting improved predictive accuracy in these regions. Adjustment for age and gender resulted in modest increases in pseudo-R^2^ and reductions in MAE across most quantiles for WC and WHtR, indicating improved model fit after covariate adjustment. In contrast, WHR showed consistently low pseudo-R^2^ values and comparatively higher MAE across quantiles, reflecting limited explanatory and predictive capacity, despite some statistically significant coefficients at higher quantiles. Overall, the combined pseudo-R^2^ and MAE results reinforce the coefficient estimates, indicating that WC and WHtR provide more consistent explanatory power and predictive accuracy for BMI across the distribution than WHR.

For WC, both the unadjusted and adjusted models showed a consistent positive association with BMI across all quantiles (*p* < 0.001). The effect size increased from Q (0.10) = 0.28 [0.25, 0.31] in the adjusted model to Q (0.90) = 0.43 [0.39, 0.47], indicating that WC has a stronger impact on BMI in individuals with higher BMI percentiles. This suggests that abdominal obesity may be more influential in individuals with higher BMI levels.

The association between WHR and BMI showed notable variations across quantiles. In the lower quantiles (Q10 and Q25), WHR had a negative association with BMI, but these effects became statistically insignificant after adjustment (*p* = 0.068, *p* = 0.457). However, at higher quantiles (Q75 and Q90), WHR showed a strong positive effect, with Q (0.75) = 14.24 [6.46, 22.01], *p* < 0.001 and Q (0.90) = 15.65 [2.07, 29.23], *p* = 0.024 in the adjusted model. This suggests that WHR becomes more relevant in predicting BMI for individuals with higher BMI values, potentially indicating a stronger impact of central obesity in overweight and obese individuals.

**Figure 1:**
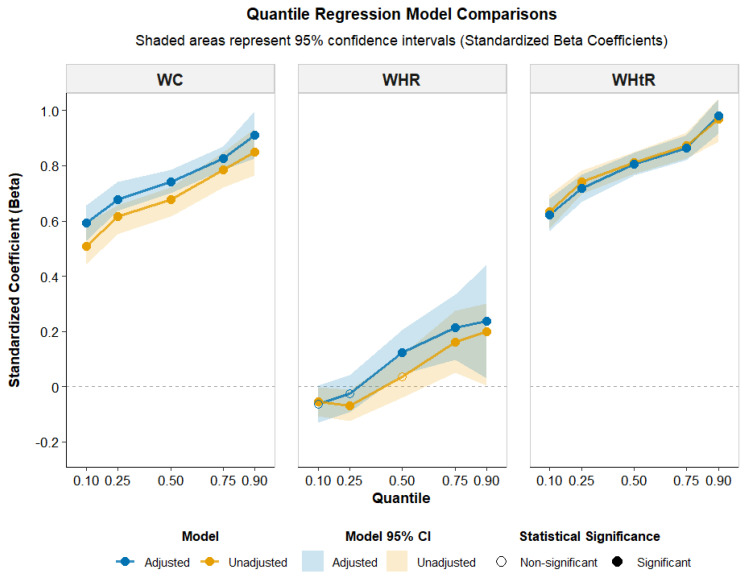
Comparisons of quantile regression models.

WHtR demonstrated consistent significant and positive association with BMI across all quantiles. In the adjusted model, the effect size increased with BMI percentiles, from Q (0.10) = 47.15 [42.75, 51.55] to Q (0.90) = 74.15 [69.35, 78.95] (*p* < 0.001 for all quantiles). The associations suggest that WHtR is also a stronger predictor of BMI across in overweight and obese individuals. Figure 1 presents standardized quantile regression coefficients. WHtR demonstrates the strongest association, with standardized coefficients approximately 0.5–0.7. Adjustment for covariates generally strengthens these associations across most quantiles, particularly for WHR at higher BMI levels. These findings suggest that the relationship between abdominal adiposity measures and BMI varies across the BMI distribution, with WHtR and WC showing the strongest and most consistent association, especially at higher BMI levels. These findings highlight the importance of considering different waist-related measures when assessing BMI and suggest that WHtR and WC are the most robust predictors across all BMI levels.

**Figure 2:**
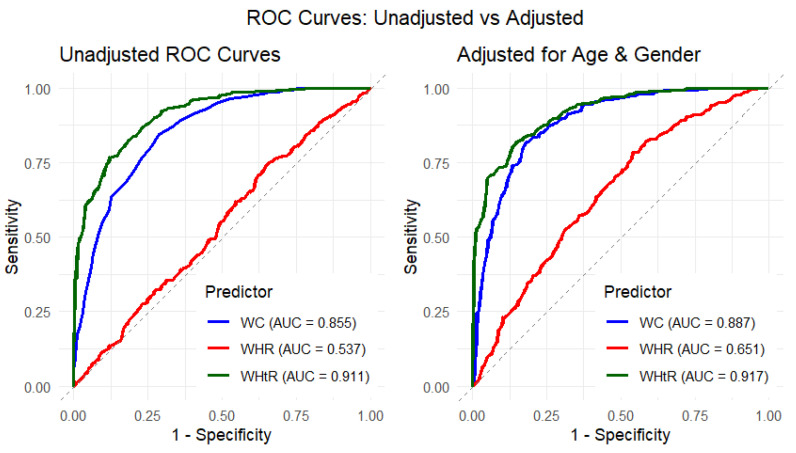
Unadjusted and adjusted ROC curves for the predictive performance of waist circumference-related measures on BMI.

The ROC analysis further supports the quantile regression findings by demonstrating clear differences in the discriminatory performance of waist circumference-related measures for BMI (Figure 2; Table 4). In the unadjusted models, WHtR showed the highest predictive accuracy, with an AUC of 0.911 (95% CI: 0.892–0.930; *p* < 0.001), followed by WC (AUC = 0.855; 95% CI: 0.828–0.882; *p* < 0.001). In contrast, WHR demonstrated poor discriminatory ability (AUC = 0.537; 95% CI: 0.495–0.578; *p* = 0.082), indicating limited capacity to distinguish BMI categories in the unadjusted analysis. After adjusting for age and gender, overall model performance improved. WHtR remained the strongest predictor (AUC = 0.917; 95% CI: 0.898–0.936; *p* < 0.001), followed by WC (AUC = 0.887; 95% CI: 0.863–0.911; *p* < 0.001). Notably, WHR showed a moderate improvement in discriminatory ability after adjustment (AUC = 0.651; 95% CI: 0.612–0.690; *p* < 0.001), although its performance remained inferior to that of WHtR and WC.

Classifier evaluation metrics were consistent with these findings. In the unadjusted models, WHtR achieved the highest Gini index (0.822) and maximum KS statistic (0.649) at a cutoff of 0.476, followed by WC (Gini = 0.711; KS = 0.558). WHR exhibited minimal predictive utility (Gini = 0.073; KS = 0.095). In the adjusted models, WHtR again demonstrated the highest discriminatory power (Gini = 0.835), followed by WC (Gini = 0.773), while WHR showed substantially lower performance (Gini = 0.301). Overall, both unadjusted and adjusted analyses indicate that WHtR and WC provide more reliable and robust discrimination of BMI variation in this population compared with WHR, reinforcing the patterns observed in the quantile regression results.

**Figure 3:**
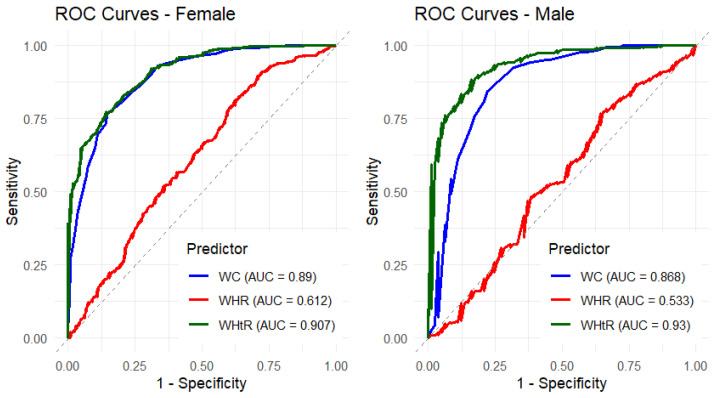
Unadjusted ROC curves by gender for the predictive performance of waist circumference-related measures on BMI.

**Figure 4:**
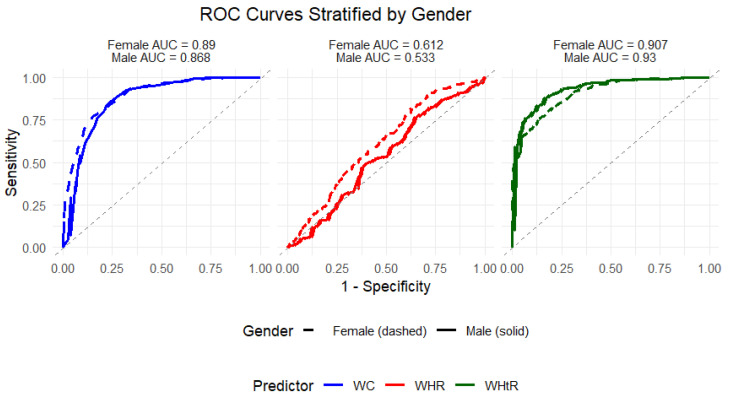
Unadjusted ROC curves stratified by gender for the predictive performance of waist circumference-related measures on BMI.

The gender-stratified ROC analyses (Figure 3 and Figure 4; Table 5) provide additional insight into the discriminatory performance of waist circumference-related measures for BMI among females and males.

Among females, WHtR demonstrated the highest predictive accuracy (AUC = 0.907; 95% CI: 0.892–0.931; *p* < 0.001), closely followed by WC (AUC = 0.890; 95% CI: 0.862–0.918; *p* < 0.001). WHR showed comparatively weaker, though statistically significant, discriminatory ability (AUC = 0.612; *p* < 0.001). The classifier evaluation metrics were consistent with these findings, with WHtR yielding the highest Gini index (0.815) and KS statistic, followed by WC (Gini = 0.780), whereas WHR demonstrated substantially lower predictive performance (Gini = 0.223).

Among males, WHtR again exhibited the strongest discriminatory performance (AUC = 0.930; 95% CI: 0.898–0.936; *p* < 0.001), with WC also showing high accuracy (AUC = 0.868; *p* < 0.001). In contrast, WHR displayed poor and non-significant discrimination (AUC = 0.533; *p* = 0.093), indicating limited usefulness for BMI classification in males. The Gini indices further reflected this pattern, with WHtR and WC demonstrating superior discriminatory capacity relative to WHR.

Comparatively, WHtR consistently outperformed both WC and WHR in both sexes, with slightly higher AUC values observed among males. WC also maintained strong performance across genders, while WHR showed modest discrimination in females and negligible predictive ability in males. Overall, the stratified analyses reinforce the robustness of WHtR and to a slightly lesser extent WC as reliable anthropometric indicators for discriminating BMI categories in both females and males within this population.

## 4. Discussion

This study examined demographic and waist-related anthropometric characteristics associated with BMI and assessed the predictive performance of WC, WHR, and WHtR across the BMI distribution using quantile regression. The findings provide important insights into the variability of central adiposity indicators as predictors of BMI in a young adult population. These results are consistent with existing literature, which emphasizes the increasing limitations of BMI as a lone measure of adiposity and cardiometabolic risk. Although BMI remains a common tool in both clinical practice and population studies due to its ease of use and low cost. However, it does not distinguish between variations in body composition, patterns of fat distribution, or levels of muscle mass [22,23]. Research increasingly demonstrates that the accumulation of visceral fat rather than total body fat is more strongly associated with metabolic conditions such as hypertension, diabetes, and cardiovascular disease [12,24]. As a result, indicators of central adiposity are now often recommended to supplement BMI when evaluating cardiometabolic risk.

The prevalence of overweight and obesity observed in this study (22.8% and 12.0%, respectively) reflects a growing public health concern, consistent with emerging trends among young adults in low- and middle-income countries [8,9]. These findings highlight the urgent need for health promotion interventions targeting physical activity, dietary behaviors, and lifestyle change among students. Previous studies in South Africa have demonstrated that tailored health-promotion programs can effectively increase self-reported physical activity and raise awareness of healthy behaviors among university students [34,35], suggesting that similar interventions could be scaled up to address the growing burden of overweight and obesity in this population. Further, the significantly higher proportions of overweight and obesity among female participants align with prior evidence showing gender disparities in adiposity, often attributed to differences in fat distribution, hormonal influences, and lifestyle factors. 

In this current study, WC showed a positive and increasingly robust association with BMI toward the upper quantiles, indicating that individuals with greater overall adiposity are more likely to accumulate abdominal fat. This pattern is consistent with previous studies showing that WC serves as a practical and reliable indicator of central adiposity and metabolic risk [24,27]. Among all indices examined, WHtR emerged as the strongest predictor. Its effectiveness may stem from its adjustment for height, which reduces misclassification linked to differences in body stature. The current literature highlights WHtR as a superior tool for identifying early cardiometabolic risk across varied populations [30,31], and the present study supports this conclusion within a young adult African cohort. This pattern suggests that WHtR captures excess adiposity more effectively in individuals with overweight and obesity, likely due to its standardization for height, which may correct for body-size-related variation not fully accounted for by WC alone. Beyond the potential underestimation of metabolic risk, an important limitation of BMI particularly in young adult populations, that is, its tendency to misclassify adiposity phenotypes, especially among individuals with higher lean mass or varying physical activity levels. This limitation is especially relevant in young adults, where substantial heterogeneity in body composition may obscure differences in central adiposity when BMI is used as a sole indicator. Recent evidence from physically active young adult populations has similarly demonstrated the limited discriminatory value of BMI alone, while highlighting the greater utility of waist-based indicators in differentiating adiposity patterns and potential metabolic risk [36]. These findings align with the present results and support the interpretation that waist-related measures provide complementary information beyond BMI in this age group.

The predictive behavior of WHR differed from WC and WHtR. While WHR showed weak or negative associations at lower BMI quantiles, its predictive strength became significant at higher quantiles [28,32]. This nonlinear pattern indicates that WHR may be less sensitive for detecting central adiposity among normal-weight individuals but may provide complementary information for distinguishing patterns of fat accumulation among those with higher BMI. However, the overall weaker predictive performance suggests that WHR may be less optimal as a standalone screening tool compared with WC and WHtR [28,32]. The multilevel insights provided by quantile regression highlight an important mehodological contribution. Unlike traditional mean-based models, quantile regression captures heterogeneity in the relationships between adiposity indicators and BMI. This allowed us to identify that waist-related metrics become more influential as individuals move up the BMI distribution. The slightly higher AUC values observed among females for WC and WHR may indicate sex-specific differences in fat distribution and body composition. Females generally store more subcutaneous adipose tissue, which may correlate more consistently with BMI classification [37,38]. In contrast, greater variability in muscle mass and visceral fat among males might weaken the predictive power of WC and WHR. Notably, WHtR showed the strongest and most consistent ability to discriminate across both sexes, confirming its reliability as a standardized measure of central adiposity. Such findings highlight the importance of adopting distribution-sensitive analytical approaches in anthropometric research, particularly when variability and skewness in adiposity measures are anticipated.

Collectively, the study’s findings affirm that WHtR and WC are strong and reliable predictors of BMI across all levels of adiposity, with WHtR showing the most pronounced increases in effect size among individuals with elevated BMI. These indicators remain simple, accessible, and cost-effective measures that could enhance early identification of individuals at risk for overweight and obesity-related health complications [30,31]. Future research should consider longitudinal designs to validate the predictive utility of these indicators over time and incorporate metabolic biomarkers to deepen understanding of associated cardiometabolic risk. Nonetheless, the current findings offer compelling evidence supporting the integration of WHtR and WC into routine anthropometric screening in young adult populations. In addition, while the present study used quantile regression to examine heterogeneity in associations across the BMI distribution, future research could employ restricted cubic splines (RCS) to model potential nonlinear relationships between WC, WHR, WHtR and BMI. This approach would allow exploration of smooth, nonlinear trends in both continuous BMI and overweight/obesity risk, complementing distribution-focused analyses and providing additional insights into the shape of these associations across the population.

## 5. Conclusions

This study demonstrates that waist-related anthropometric indicators, particularly WHtR and WC, show consistent and quantile-specific associations with BMI in young adults. In contrast, WHR exhibited weaker and less consistent relationships across the BMI distribution, suggesting limited utility relative to WHtR and WC within BMI-based frameworks. The findings highlight heterogeneity in the relationships between central adiposity measures and BMI across different levels of BMI and underscore the potential value of WHtR and WC as complementary anthropometric measures in population-based assessments. Using the ROC curve analyses, both unadjusted and adjusted analyses indicate that WHtR and WC provide more reliable and robust prediction of BMI variation in this population compared with WHR, reinforcing the patterns observed in the quantile regression results. Furthermore, WHtR consistently demonstrated superior prediction performance compared with both WC and WHR in males and females, with slightly higher accuracy observed among males. WC also maintained strong and stable predictive ability across genders, whereas WHR showed only modest discrimination in females and negligible predictive performance in males. Collectively, these findings highlight the value of WHtR and, to a slightly lesser extent, WC as practical and dependable anthropometric indicators for BMI classification in this population. These results should be interpreted within the context of BMI as a proxy outcome, and further research incorporating direct measures of adiposity is warranted to extend the present findings.

## 6. Limitations

The study population consists predominantly of young adults, which may limit generalizability to older age groups or diverse demographic settings. Additionally, a key limitation of this study is the absence of direct measures of body composition, such as body fat percentage or dual-energy X-ray absorptiometry, which restricted the use of BMI as the reference outcome. While BMI is widely used in epidemiological and clinical settings, it does not distinguish between fat and lean mass and therefore may not fully capture adiposity-related risk. Therefore, future studies should incorporate direct measures of body composition to further evaluate and validate the predictive performance of waist circumference-related measures beyond BMI-based frameworks. Further, the cross-sectional design limits causal inference, hence future research incorporating longitudinal follow-up would strengthen understanding of these anthropometric relationships over time. Participation in the study was based on voluntary enrolment, followed by random selection from the pool of respondents. Although this approach helps to reduce selection bias, the initial voluntary participation may still be associated with health-related self-selection, which could limit the generalizability of the findings. Lastly, data on physical activity were collected but were not incorporated into the analyses presented in this paper. As physical activity may influence BMI, this should be considered when interpreting the findings.

## Figures and Tables

**Table 1:** Demographic characteristics and anthropometric measurements of the sampled participants.

Characteristic	*N* (%) or Mean ± SD
Gender	
*Male*	305 (36.2)
*Female*	537 (63.8)
Age (in years)	21.78 ± 4.04
Weight (in kg)	66.42 ± 14.94
Height (in cm)	166.45 ± 9.19
Gluteal (in cm)	102.37 ± 12.22
BMI (in kg/m^2^)	24.0 ± 5.3
Waist Circumference (in cm)	79.85 ± 11.24
Waist Hip Ratio	0.78 ± 0.08
Waist-to-Height Ratio	0.48 ± 0.07
BMI Categories	
*Underweight*	70 (8.3)
*Normal weight*	479 (56.9)
*Overweight*	192 (22.8)
*Obese*	101 (12.0)

Note: *N* = 842.

**Table 2:** Comparisons and associations of demographic characteristics and waist circumference-related measures with BMI.

Characteristic	Underweight *N* = 70	Normal Weight *N* = 479	Overweight *N* = 192	Obese *N* = 101	*p*-Value
	N (%) or Mean ± SD	N (%) or Mean ± SD	N (%) or Mean ± SD	N (%) or Mean ± SD	
Gender					
*Male*	35 (11.5)	189 (62.0)	62 (20.3)	19 (6.2)	<0.001 *
*Female*	35 (6.5)	290 (54.0)	130 (24.2)	82 (15.3)	
Age (in years)	21.03 ± 3.63 ^(a)^	21.27 ± 3.46 ^(a,b)^	22.52 ± 4.41 ^(b,c)^	23.28 ± 4.04 ^(c)^	<0.001 *
Weight (in kg)	50.41 ± 4.81 ^(a)^	60.52 ± 8.00 ^(b)^	73.47 ± 9.47 ^(c)^	92.13 ± 16.56 ^(d)^	<0.001 *
Height (in cm)	170.15 ± 7.68 ^(a)^	167.29 ± 8.63 ^(a,b)^	164.76 ± 9.25 ^(b,c)^	163.08 ± 10.99 ^(c)^	<0.001 *
Gluteal (in cm)	87.86 ± 5.25 ^(a)^	97.81 ± 7.51 ^(b)^	108.86 ± 8.21 ^(c)^	121.67 ± 11.62 ^(d)^	<0.001 *
WC (in cm)	70.30 ± 7.61 ^(a)^	75.59 ± 7.30 ^(b)^	84.77 ± 8.67 ^(c)^	97.26 ± 10.98 ^(d)^	<0.001 *
WHR	0.80 ± 0.09 ^(a)^	0.78 ± 0.07 ^(a,b)^	0.78 ± 0.08 ^(b)^	0.80 ± 0.08 ^(b)^	0.002 *
WHtR	0.41 ± 0.04 ^(a)^	0.45 ± 0.04 ^(b)^	0.51 ± 0.04 ^(c)^	0.60 ± 0.06 ^(d)^	<0.001 *

(*) Statistically significant differences at Alpha = 0.05. Note: Tukey’s HSD test was used for post hoc comparisons. Means within a row that do not share a common superscript letter (a–d) differ significantly (*p* < 0.05), with a denoting the lowest and d the highest mean.

**Table 3:** Unadjusted and adjusted quantile regression estimates for the predictive effect of waist circumference-related measures on BMI.

Measure	Unadjusted Model			^a^ Adjusted Model		
	Coefficient [95% CI]	R^2^ [MAE]	*p*-Value	Coefficient [95% CI]	R^2^ [MAE]	*p*-Value
Predictor Variable: WC						
Q (0.10)	0.24 [0.21, 0.28]	0.164 [4.26]	<0.001 *	0.28 [0.25, 0.31]	0.231 [3.74]	<0.001 *
Q (0.25)	0.29 [0.26, 0.31]	0.261 [3.04]	<0.001 *	0.32 [0.30, 0.35]	0.331 [2.77]	<0.001 *
Q (0.50)	0.32 [0.29, 0.34]	0.328 [2.59]	<0.001 *	0.35 [0.33, 0.37]	0.402 [2.31]	<0.001 *
Q (0.75)	0.37 [0.34, 0.40]	0.377 [3.19]	<0.001 *	0.39 [0.37, 0.41]	0.456 [2.79]	<0.001 *
Q (0.90)	0.40 [0.36, 0.44]	0.407 [4.57]	<0.001 *	0.43 [0.39, 0.47]	0.461 [3.92]	<0.001 *
Predictor Variable: WHR						
Q (0.10)	−3.59 [−7.06, −0.11]	0.005 [5.46]	0.043 *	−4.15 [−8.60, 0.30]	0.019 [5.43]	0.068
Q (0.25)	−4.53 [−8.32, −0.73]	0.005 [4.56]	0.020 *	−1.67 [−6.07, 2.73]	0.034 [4.37]	0.457
Q (0.50)	2.44 [−2.69, 7.57]	0.001 [3.86]	0.351	8.20 [2.89, 13.52]	0.053 [3.66]	0.003 *
Q (0.75)	10.75 [3.26, 18.24]	0.010 [4.75]	0.005 *	14.24 [6.46, 22.01]	0.064 [4.52]	<0.001 *
Q (0.90)	13.16 [0.30, 20.01]	0.014 [7.55]	0.045 *	15.65 [2.07, 29.23]	0.066 [7.13]	0.024 *
Predictor Variable: WHtR						
Q (0.10)	47.96 [43.38, 52.53]	0.253 [3.63]	<0.001 *	47.15 [42.75, 51.55]	0.267 [3.56]	<0.001 *
Q (0.25)	56.05 [52.75, 59.35]	0.359 [2.59]	<0.001 *	54.44 [50.73, 58.15]	0.366 [2.57]	<0.001 *
Q (0.50)	61.34 [58.31, 64.38]	0.441 [2.16]	<0.001 *	61.02 [57.88, 64.16]	0.454 [2.11]	<0.001 *
Q (0.75)	66.13 [62.66, 69.61]	0.499 [2.58]	<0.001 *	65.53 [62.02, 69.01]	0.513 [2.52]	<0.001 *
Q (0.90)	73.31 [67.25, 79.01]	0.523 [3.75]	<0.001 *	74.15 [69.35, 78.95]	0.541 [3.70]	<0.001 *

(*) Statistically significant at Alpha = 0.05. ^a^: Model is adjusted by gender and age. CI is Confidence Interval, R^2^ is Pseudo R Squared and MAE is the Mean Absolute Error.

**Table 4:** Area under the unadjusted and adjusted curves (AUC) for the predictive performance of waist circumference-related measures on BMI.

Variable(s)	Area	Std. Error	*p*-Value	Asymptotic 95% Confidence Interval		Classifier Evaluation Metrics	
				Lower Bound	Upper Bound	Gini Index	Max KS [Cutoff]
Unadjusted							
WC	0.855	0.014	<0.001 *	0.828	0.882	0.711	0.558 [82.000]
WHR	0.537	0.021	0.082	0.495	0.578	0.073	0.095 [0.821]
WHtR	0.911	0.010	<0.001 *	0.892	0.930	0.822	0.649 [0.476]
Adjusted by Age and Gender							
WC	0.887	0.006	<0.001 *	0.863	0.911	0.773	1.639 [0.651]
WHR	0.651	0.010	<0.001 *	0.612	0.690	0.301	1.246 [0.605]
WHtR	0.917	0.005	<0.001 *	0.898	0.936	0.835	1.673 [0.695]

(*) Statistically significant at Alpha = 0.05. KS is the Kolmogorov–Smirnov Metric.

**Table 5:** Area under the curve (AUC) by gender for the predictive performance of waist circumference-related measures on BMI.

Variable(s)	Area	Std. Error	*p*-Value	Asymptotic 95% Confidence Interval		Classifier Evaluation Metrics	
				Lower Bound	Upper Bound	Gini Index	Max KS [Cutoff]
Females							
WC	0.890	0.007	<0.001 *	0.862	0.918	0.780	1.620 [77.500]
WHR	0.612	0.013	<0.001 *	0.495	0.661	0.223	1.193 [0.790]
WHtR	0.907	0.006	<0.001 *	0.892	0.931	0.815	1.631 [0.475]
Males							
WC	0.868	0.013	<0.001 *	0.863	0.911	0.920	1.622 [85.500]
WHR	0.533	0.020	0.093	0.612	0.690	0.610	1.126 [0.869]
WHtR	0.930	0.008	<0.001 *	0.898	0.936	0.962	1.723 [0.494]

(*) Statistically significant at Alpha = 0.05. KS is the Kolmogorov-Smirnov Metric.

## Data Availability

The data supporting the findings of this study are available from the corresponding author upon reasonable request.
